# Effect of malaria on productivity in a workplace: the case of a banana plantation in Zimbabwe

**DOI:** 10.1186/s12936-019-3021-6

**Published:** 2019-12-03

**Authors:** Akim Tafadzwa Lukwa, Richard Mawoyo, Karen Nelwin Zablon, Aggrey Siya, Olufunke Alaba

**Affiliations:** 1 Health Economics Unit, School of Public Health and Family Medicine, Faculty of Health Sciences, University of Cape Town, Anzio Road, Observatory, Cape Town, 7925 South Africa; 2 Mutare Provincial Hospital, P. O. Box 30, Mutare, Zimbabwe; 3 National Institute for Medical Research, P.O Box 1462, Mwanza, Tanzania; 4 College of Veterinary Medicine, Animal Resources and Biosecurity, Makerere University, P.O. Box 7062, Kampala, Uganda

**Keywords:** Malaria, Absenteeism, Productivity, Agriculture

## Abstract

**Background:**

Malaria is known to contribute to reduction in productivity through absenteeism as worker-hours are lost thus impacting company productivity and performance. This paper analysed the impact of malaria on productivity in a banana plantation through absenteeism.

**Methods:**

This study was carried out at Matanuska farm in Burma Valley, Zimbabwe. Raw data on absenteeism was obtained in retrospect from the Farm Manager. Malaria infection was detected using malaria Rapid Diagnostic Test. Measures of absence from work place were determined and included; incidence of absence (number of absentees divided by the total workforce), absence frequency (number of malaria spells), frequency rate (number of spells divided by the number of absentees), estimated duration of spells (number of days lost due to malaria), severity rate (number of days lost divided by number of spells), incapacity rate (number of days lost divided by the number of absentees), number of absent days (number of spells times the severity rate), number of scheduled working days (actual working days in 5 months multiplied by total number of employees), absenteeism rate.

**Results:**

A total of 143 employees were followed up over a 5-month period. Malaria positivity was 21%, 31.5%, 44.8%, 35.7% and 12.6% for January 2014 to May 2014, respectively. One spell of absence [194 (86.6%)] was common followed by 2 spells of absence [30 (13.4%)] for all employees. Duration of spells of absence due to malaria ranged from 1.5 to 4.1 working-days, with general workers being the most affected. Incidence of absence was 143/155 (93.3%), with total of spells of absence of over a 5-month period totalling 224. The frequency rate of absenteeism was 1.6 with severity rate of absence being 2.4. and incapacity rate was 3.7.

**Conclusion:**

Malaria contributes significantly to worker absenteeism. Employers, therefore, ought to put measures that protect workers from malaria infections. Protecting workers can be done through malaria educative campaigns, providing mosquito nets, providing insecticide-treated work suits, providing repellents and partnering with different ministries to ensure protection of workers from mosquito bites.

## Background

Globally, malaria affects about 200 million people undermining their productivity at household, national and international scales [1]. In sub-Saharan Africa, where agriculture is the main source of livelihood, malaria accounts for the biggest proportion of deaths compared to other diseases associated with agriculture [1]. Sub-Saharan Africa is known to have some of the poorest human population in the world, with fifteen countries in sub-Saharan Africa and India carrying almost 80% of the global malaria burden [2]. Nearly half of all malaria cases worldwide can be attributed to five countries: Nigeria (25%), Democratic Republic of the Congo (11%), Mozambique (5%), India (4%) and Uganda (4%) [2]. In Zimbabwe, indoor residual spraying (IRS) and long-lasting insecticidal nets (LLINs) are some of the vector control methods used in fighting against malaria. The National Malaria Control Programme (NMCP) coordinates IRS in eight malaria-endemic provinces (Manicaland, Mashonaland East, Mashonaland West, Mashonaland Central, Masvingo, Matebeleland North, Matebeleland South, Midlands). Dichlorodiphenyltrichloroethane (DDT), pyrethroids and an additional organophosphate insecticide (pirimiphos-methyl) are the insecticides used for IRS in Zimbabwe [3].

Malaria transmission is influenced by climatic conditions that affect the survival of mosquitoes, such as rainfall patterns, temperature and humidity. Due to varying rainfall patterns, temperature and humidity malaria transmission is usually seasonal with peaks often experienced during and immediately following the rainy season [2, 4]. Malaria epidemics usually increase when, among other factors, climatic conditions favour transmission of malaria which is dominant in areas where people have little or no immunity to malaria [2].

It is known that human health plays a key role in determining productivity at both household and country scales [5]. Diseases like malaria which undermine human health and wellbeing often reduce productivity of those affected directly and indirectly [6–8].

Due to the severity of malaria, once someone is affected, they always seek treatment and sometimes a break from work [9] which is a significant concern for agricultural productivity [10]. Absence from work results in loss of significant proportion of income or earnings [11], in instances where organisations do not compensate earnings during sick days. In agricultural sector, the majority of workers are paid based on output or production, therefore, medical expenses incurred in seeking treatment and earnings lost translate into economic cost associated with disease transmission [12]. Assessment of the impact of absenteeism heavily relies on the number of spells of absence as the latter indicator gives a reflection of the magnitude of the problem, irrespective of the reason for absenteeism [13].

A study done in Côte d’Ivoire revealed that cabbage farmers were sick for 14–15 days from different diseases, however malaria accounted for 8–9 days of absent days thus constituting 58% of the sick days compared with other diseases [11]. In Southeast Ethiopia, absenteeism due to sickness among horticultural workers accounted for 58.8% of all days lost, with malaria accounting for 23.6% of the lost days [14]. In a Côte d’Ivoire study, a correlation between absenteeism, crop yields and revenue was observed with decreasing crop production attributed to frequent farmers absenteeism due to sickness [11]. Results showed that farm workers that had been sick for more than 2 days (mean: 4.2 days) had 47% lower yields and 53% lower revenues than farmers who missed a maximum of 2 days (mean: 0.3 days) [11]. In countries like Zimbabwe, which lie in the malaria belt of Africa, there is a dearth of information on the effect of malaria on productivity of farm workers despite agriculture being the main economic activity.

This study sought to elucidate the impact of malaria on productivity in a banana plantation through absenteeism reducing the labour units on hand and, therefore, reducing overall farm production.

## Methods

### Study area

**Fig. 1:**
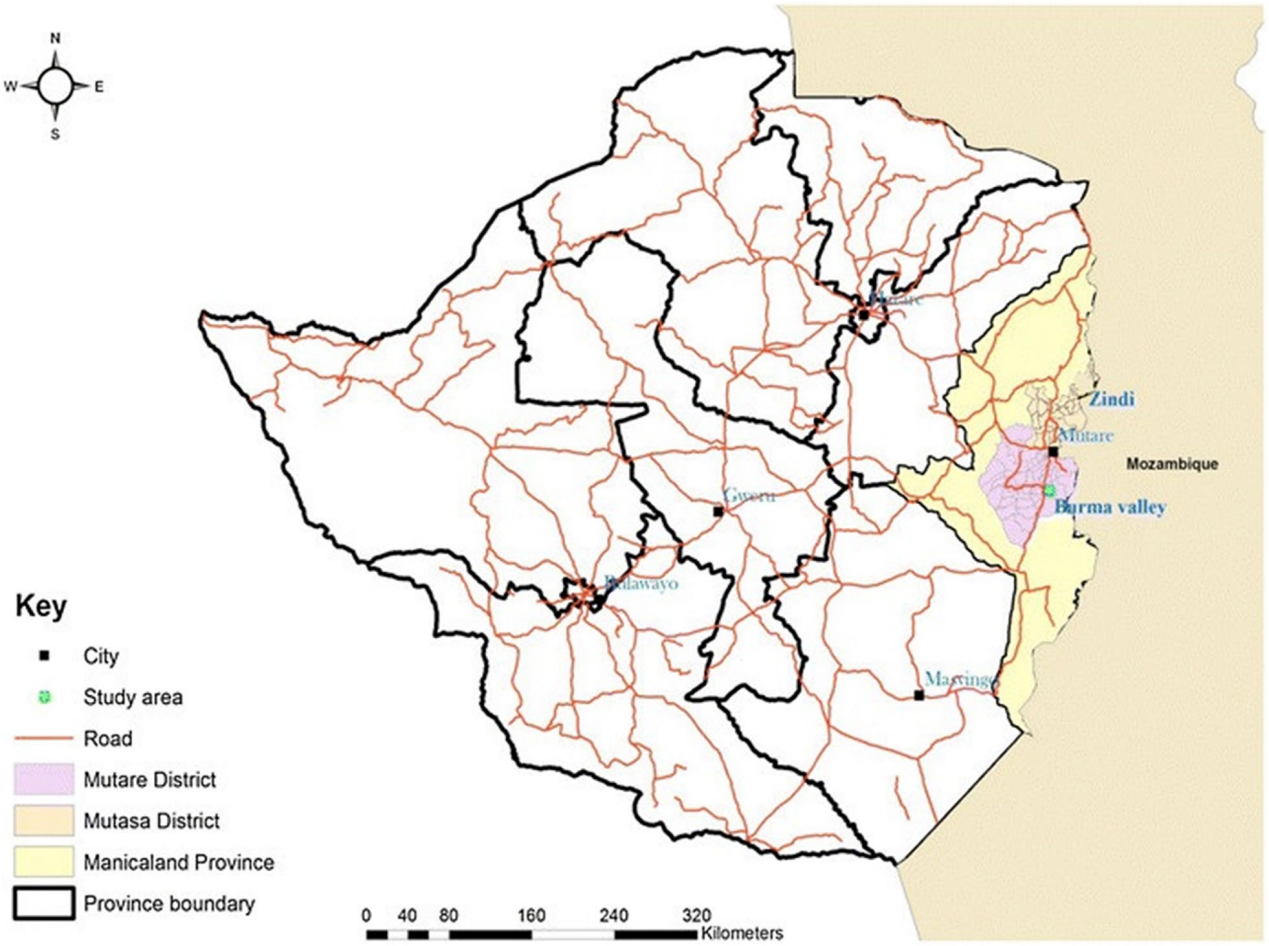
Map of the study area

This study was conducted in Manicaland province, Zimbabwe. Manicaland province has 7 districts, namely Buhera, Chimanimani, Chipinge, Makoni, Mutare, Mutasa and Nyanga and 3 major valleys (Burma, Sabi and Honde) (Fig. 1). This study was conducted at Matanuska farm (18° 97′ 055′′ E, 32° 67′ 083′′ S), which is in Mutare district in Zimbabwe.

### Malaria transmission in the area

Matanuska farm is situated in Burma Valley which is in Mutare district. Manicaland province has a highly mobile population that serves timber, tea and banana plantations and subsistence farmers grow bananas, avocadoes, yams and maize. The major malaria vector in Manicaland province is *Anopheles funestus* sensu stricto and has been observed to be resistant to pyrethroids [15]. At the time of the study, the area was implementing an IRS strategy using an organophosphate insecticide. For malaria treatment country wide, Zimbabwe uses the artemisinin-based combination therapy (ACT) [16].

### Agricultural activities in the study area

Matanuska farm specializes in banana farming all year round and it is served by Burma Valley Health Centre which is 7 km from the farm. However, patients with complications were referred to Mutare Provincial hospital. All malaria cases were tested at the farm by Village Health Workers (VHW) using the malaria rapid diagnostic test (RDT) kit. All employees who were RDT positive were treated using ACT and referred to the farm manager who grants 3-days sick leave (inclusive of non-working days if they are part of the 3 sick leave days).

### Farm workers

Matanuska farm has permanent employees, but also employs casual workers during peak banana farming seasons. This study will focus on the permanent employees employed from January to May of 2014 as from the data obtained permanent employees had complete data on the attributes under study. The farm employed 155 permanent workers in the months of January, February, March, April and May respectively. A total of 143 out of 155 (92.3%) permanent workers were followed up during the study looking at the availed data in retrospect. Of the 143 permanent workers followed up they were; 92 general workers, 6 workshop personnel, 22 security guards, 9 supervisors, 7 administration, 6 drivers and 1 Village Health Worker, respectively (Table 1).

**Table 1:** Distribution of worker categories, January to May 2014

Worker category	Frequency	Percentage	Cumulative
General workers	92	64.34	64.34
Workshop personnel	6	4.2	68.53
Guards	22	15.38	83.92
Supervisors	9	6.29	90.21
Administration	7	4.90	95.10
Drivers	6	4.20	99.30
VHW	1	0.70	100.00
Total	143	100.00	

### Computation of incidence of absenteeism due to malaria among farm workers

Incidence of absenteeism due to malaria for each of the 7 worker categories in this study was defined as ‘the number of days lost within one spell divided by the total number of days required to work. For each spell of absence (worker had an RDT positive result) a worker was given time off in all the worker categories while spell of absence was defined as days lost due to malaria in relation to malaria episodes encountered (Table 2).

**Table 2:** Measures of absenteeism due to malaria. Adopted from [13]

Parameter	How it was determined
Incidence of absence	Is the number of days lost in one spell of the worker category divided by total days of that worker category × 100
Absence frequency	Total number of spells in 5 months
Absenteeism frequency rate	Average number of spells per absentee (total number of malaria episodes divided by total number of absentees)
Total duration (in days) of spells of absenteeism	Total number of days lost due to malaria
Severity rate	Average duration of spells (total number of days lost due to malaria divided by total number of malaria episodes)
Incapacity rate	Mean number of days lost per absentee (total number of days lost due to malaria divided by the number of absentees)
Number of listed public holidays	These were calculated from the calendar for each month as gazetted by the Government of Zimbabwe
Number of working days	All working days (5.5 days per week) were added up for 5 months
Total number of absent days by workers	Absence frequency × average duration of spells (spells × severity rate)
Total number of scheduled working days for all workers	All working days excluding holidays in 5 months × total workers
Absenteeism rate	(Total number of absent days divided by total scheduled days) × 100

### Data collection

Malaria was detected using RDTs, performed by Village Health Workers (VHW) at the farm. All employees who were RDT positive were treated using ACT. The RDT used for testing malaria in this study was Paracheck-Pf^®^, since 98% of malaria in Zimbabwe is *Plasmodium falciparum* [15]. The study data was then used to assess malaria transmission and absenteeism rates in permanent workers belonging to the following categories: administration staff, supervisors, workshop personnel, drivers, general workers, guards and VHW. However, VHWs are not financially compensated on malaria activities in Zimbabwe. For this study; January (24 days), February (22 days), March (23.5 days), April (23 days) and May (23.5 days) are the working days consider for the period under review.

### Methods used to measure absenteeism

#### Data handling and analysis

Data were obtained from the Farm Manager in an Excel spreadsheet. The excel sheet had data on observational number, age, gender, malaria status, job title and number of days worked. Since there were no names or identifying markers on the availed data, findings cannot not be traced to specific individuals. A descriptive analysis was done using Analysis of variance (ANOVA) in excel and STATA version 14.

## Results

Overall malaria positivity rates for the period were January 23% (24% females; 76% males), February 32% (39% females; 61% males), March 45% (25% females; 75% males), April 34% (31% females; 69% males) and May 13% (21% females; 79% males). In 2014, overall malaria transmission across worker categories at Matanuska farm increased gradually in January (23%) and February (32%) reaching a peak in March (45%) before a gradual decline in April (34%) and May (13%).

### Characteristics of workers

Males constituted most workers whose ages were not significantly different from females (p ≥ 0.3) despite the fact the age range was higher in males than females, where mean ages were; males (33.8, SD = 9.8) and females (35.9, SD = 9) (Table 3).

**Table 3:** Sex distribution of employees (N = 143), January to May 2014

	N	Age of employees (years)			
		Minimum	Maximum	Range	Mean
Males	97 (67.8%)	20	64	44	33.8 ± 9.8
Females	46 (32.2%)	17	58	41	35.9 ± 9

### Malaria positivity rate

**Fig. 2:**
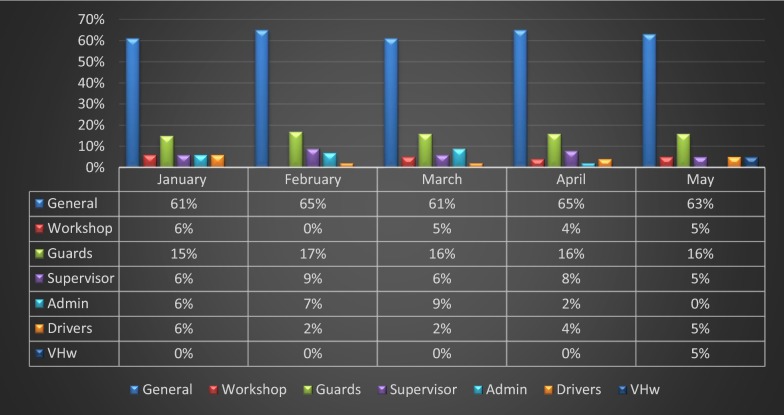
Malaria positivity across worker categories, January to May 2014

All categories of permanent employees had malaria episodes at any one time during the 5-months period (Fig. 2). In the study there was only 1 VHW and the VHW only had a malaria episode once in May.

### Incidence of absenteeism due to malaria among farm workers

For one spell of absence across all worker categories 86.6% lost productive days due to malaria while for two spells of absence 13.4% lost productive days due to malaria (Table 4). Spells of absence appeared to be work related as general workers had more spells of absence due to malaria compared to other worker categories and all worker categories were significantly different (p = 0.001).

**Table 4:** Incidence of absenteeism due to malaria among farm workers, January to May 2014

	Spells (episodes) of absence due to malaria		
	One spell	Two spells	Total spells of absence
General workers	130 (87.8%)	18 (12.2%)	148 (100.0%)
Workshop personnel	7 (100.0%)	0 (0.0%)	7 (100.0%)
Guards	27 (77.1%)	8 (22.9%)	35 (100.0%)
Drivers	6 (75.0%)	2 (25.0%)	8 (100.0%)
Administration staff	13 (100.0%)	0 (0.0%)	13 (100.0%)
Supervisors	10 (83.3%)	2 (16.7%)	12 (100.0%)
VHW	1 (100.0%)	0 (0.0%)	1 (100.0%)
Total	194 (86.6%)	30 (13.4%)	224 (100.0%)

### Duration of spells of absence (days) due to malaria

General workers were the major contributors (68.3%) of lost days due to malaria while 1 VHW contributed the least (0.3%) (Table 5). The mean number of lost days due to malaria was 4.1 days and 4.0 days for general workers and drivers, respectively. Excluding the one VHW worker, workshop personnel had the least mean days with 2.5 days. The number of lost days due to malaria were significantly different for all categories (p = 0.001), therefore, mean numbers of days lost due to malaria were related to the worker category and the nature of work.

**Table 5:** Days lost due to malaria among farm workers, January to May 2014

	Total estimated duration of spells (total days lost due to malaria)	% contribution by each category of workers	Mean days lost due to malaria
General workers	362.5	68.3	4.1
Workshop personnel	17.5	3.3	2.5
Guards	69.5	13.1	3.0
Drivers	24.0	4.5	4.0
Administration staff	27.0	5.1	3.4
Supervisors	29.0	5.4	3.2
VHW	1.5	0.3	1.5
Total	531.0	100.0	3.7

### Absence measures among worker categories

The incidence of absences of general workers was highest than all the other worker categories. However, results were not statistically significant for all worker categories (p = 0.37) (Table 6). Absence frequency was highest among general workers and least among workshop personnel. However, absence frequency was not statistically significant for all worker groups (p = 0.25). General workers recorded the highest frequency rate of absenteeism (1.7) and workshop personnel had the least (1.0) (Table 6). Absence frequency (total number of spells in 5-months) was statistically significant for all worker groups (p = 0.026). General workers had the longest total estimated duration of spells of absence (364.9 days) while workshop personnel recorded the shortest total estimated duration of spells of absence (17.5 days). Total estimated duration of spells of absence were not statistically significant for all worker groups (p = 0.29). The average duration of spells (severity rate of absence) was highest among drivers (3.0 days) while for general workers, workshop personnel & supervisors was the same (2.5 days) and guards & administration recorded severity rate of absence of 2.4 days (Table 6). Severity rate of absence was not statistically for all worker categories (p = 0.15). The highest frequency rate of absenteeism was recorded in general workers with 148 days of absenteeism due to malaria (Table 6).

**Table 6:** Calculated absence measures among worker categories, January to May 2014

Absence measure	General workers	Workshop personnel	Guards	Drivers	Administration staff	Supervisors	All employees
Incidence of absence (absentees/total workforce)	88.1%	100.0%	100.0%	100.0%	100.0%	100.0%	93.3%
Absence frequency (total number of spells in 5 months)	148	7	35	8	13	12	224
Frequency rate (spells/absentees)	1.7	1.0	1.5	1.3	1.6	1.3	1.6
Total estimated duration of spells (total days lost due to malaria)	364.9 days	17.5 days	69.0 days	24.0 days	27.2 days	28.8 days	531.0 days
Severity rate (average duration of spells) (days lost divided by number of spells)	2.5 days	2.5 days	2.0 days	3.0 days	2.1 days	2.4 days	2.4 days
Incapacity rate (total days lost divided by absentees)	4.1 days	2.5 days	3.0 days	4.0 days	2.1 days	2.4 days	3.7 days
Number of working days in 5 months at 5.5 days per week for all categories	116 days	116 days	116 days	116 days	116 days	116 days	116 days
Estimated total number of absent days by workers (number of spells × severity rate)	370 days	17.5 days	70 days	24 days	27.3 days	28 days	537.6 days
Total number of scheduled working days for all workers (working days in 5 months × total employees)	10,324 days	812 days	2668 days	696 days	1508 days	1392 days	17,980 days
Absenteeism rate {(total number absent days divided by total scheduled days)	3.58%	2.16%	2.62%	3.45%	1.81%	2.01%	2.99%

## Discussion

Absenteeism is the failure to report for work as scheduled regardless of the reason [17], therefore, this paper discusses permanent worker absenteeism due to malaria at a banana farm in Zimbabwe. Worker characteristics were essential in explaining variations observed during the study and how such attributes impacted on worker absenteeism in relation to malaria transmission as it was mostly work related. Most general workers work at night hence, exposed to vector mosquitoes that transmit malaria even though they sleep in sprayed houses and mosquito nets during the day when mosquitoes are not highly active. The aforementioned explains the major contribution by general workers to the lost days due to malaria.

Overall, there were 2 male workers to every female worker, which likely reflects the company recruitment policies, however their ages were not significantly different. Most of the work involved in banana production is labour intensive (maintaining plants, harvesting bananas, packing them in 15 kg crates and loading them in tractors to the processing sheds) and this is done by males within the general worker category who were the majority employees. All permanent workers and their dependents who resided at the farm were provided with long-lasting insecticide-treated mosquito nets (LLINs) through the NMCP, an arm of government.

Malaria incidence peaked in the months of March and April across all worker categories. The peak in malaria transmission can be partly attributed to existence of breeding sites, since there were a variety of vector breeding sites in the nearby communities within flying distance of vector mosquitoes [15]. A mosquito knows no geographical or social demarcations, therefore, the occurrence of malaria transmission in the nearby communities would result in local casual workers likely carrying the malaria parasites with them [18]. Although malaria transmission in our study was lower compared to a study done in South-East Ethiopia [14], there is need to invest in methods to discourage breeding sites within areas surrounding Matanuska farm.

In this study, absence from work caused by an episode of malaria attack was confirmed by an RDT positive result. In general, 86.6% of all workers experienced one (1) spell of absence due to malaria alone and this is figure is higher than 53% recorded for all absence in Nigeria [13]. Such high levels of absenteeism translate into higher costs of production due to workers being absent due to malaria [19]. The worker categories that had 1 spell of absence due to malaria were attacked once in the 5-month period, the latter implies that their exposure to malaria risk was minimal and the malaria risk exposure was work related. For 1 spell of absence the study results were higher than those recorded in Nigeria [13] and concurred with those recorded in Ethiopia [14]. However, the results were lower than that observed at the teaching hospital [20]. The study reflected that malaria had a great impact on worker attendance even when an employee was attacked by malaria once. Two spells of absence were observed with general workers, guards, drivers and supervisors. Drivers experienced twice as much 2 spells of absence as general workers due to the fact that not all general workers were always present during night shifts since they had programmed rotations. On the other hand, there were very few drivers and all of them were on frequent rotations hence this made them go through night shifts frequently. Such multiple malaria episodes reduced labour supply significantly in Nepal [21].

All workshop personnel, guards, drivers, administration staff and supervisors were absent due to malaria at any one time during the study. On the other hand, general workers had an incidence of absence of 88.1% and the general incidence of absence for all employees was 93.3%. All the observed incidences of absences across all worker categories were very high and reduced the available labour force as observed in Côte d’Ivoire [11]. However, there was no significance difference across all worker categories.

Absence rate for all employees across categories was 2 times lower than that recorded in Nigeria [13]. For the Nigerian study data was collected from 251 employees [13], while this study that looked at 143 employees. Results from this study show no significance difference over worker categories.

In general, the frequency rate of absenteeism by all workers was 1.6, implying that one worker was capable of being absent from work due to malaria 1.6 times more when compared to those who were not sick. This trend was also observed with general workers, guards, supervisors, drivers and administration staff. This was least observed with workshop personnel. Results from this study were lower than those observed in Nigeria and Ethiopia respectively [13, 14], with results showing that the frequency rate of absenteeism was significantly different over worker categories which translates to malaria being a significant contributor to absenteeism at the farm.

The general severity rate of absenteeism was 2.4 days (average duration of spells) and this was almost the same that was observed in general workers, workshop personnel and supervisors. However, the severity rate of absenteeism was almost the same with guards and administration staff but higher in drivers. The severity rates in all categories were lower than those recorded in other studies [13, 14, 20]. Workers in this study did not experience severe malaria in contrast with observations made in Nigeria [13]. The average duration of spells in our study was 0.6 times less than that observed in Nigeria [13]. The severity rate of absenteeism observed in this study was not significantly different over worker categories.

This reflects to what level an employee was incapacitated due to the absence from work. The incapacity rate of workshop personnel, supervisors and administration staff was almost the same but 2.88 times lower than that observed in Nigeria [13]. The incapacity rates of drivers and general employees were 1.8 and 1.9 times lower than those observed in Nigeria [13]. On the other hand, the incapacity rate of guards was 2.4 times lower than that observed in Nigeria [13].

Frequent absenteeism inconveniences supervisors as they have to re-assign duties each day one person is absent, making some employees uncomfortable with such kind of arrangement. The overall absenteeism rate in this study was 2.99% (about 3 employees in every 10) as compared to 1.7% (about 2 employees in every 10) in the Nigerian study [13], thus this study has 1.29 times more on the overall absenteeism rate compared to this study in Nigeria. Absenteeism rate by worker category; 3.58% for workshop personnel (4 employees in 10), 2.62% for guards (3 employees in 10), 3.45% for drivers (4 employees in 10), 1.81% for administration staff (2 employees in 10) and 2.01% for supervisors (2 employees in 10) was higher than that observed [13].

These results showed that malaria transmission severely contributed to absenteeism at Matanuska farm. Personal protection methods are encouraged, whether funded by the farm owner or individual employees. Results for all employees are closer to the extreme severe rate of 4% compared to the Nigerian study [13]. Matanuska farm management does not deduct a proportion of wages when an employee is sick, implying that the farm absorbs the costs associated with absenteeism. The findings of this study where mainly argued relative to the Nigerian study due to similar set ups.

## Conclusion

Although the final absenteeism rate was 2.99% (below the 4% excessive rate), malaria contributed significantly to worker absenteeism, to the extent that employers are strongly encouraged to put measures that protect their workers.

## Data Availability

Data is available upon request from the corresponding author

## Abbreviations

RDTs: Rapid Diagnostic Test Kits
ACT: artemisinin-based combination therapy
DDT: dichlorodiphenyltrichloroethane
LLINs: long-lasting insecticidal nets
NMCP: National Malaria Control Programme
VHW: Village Health Worker
